# Case report: Central venous catheter thrombosis complicated by chronic thromboembolic disease/pulmonary hypertension in two children requiring parenteral nutrition

**DOI:** 10.3389/fcvm.2023.1198204

**Published:** 2023-06-08

**Authors:** Maja Hanuna, Joseph Pattathu, Joscha Buech, Christine Kamla, Nikolaus Kneidinger, Juergen Behr, Katrin Milger, Tobias Veit, Marina Nagel, Jan Abicht, Robert Dalla-Pozza, Marcus Fischer, Andre Jakob, Matthias Hermann, Rene Schramm, Laura L. Rosenthal, Nikolaus Haas, Jürgen Hörer, Christian Hagl, Sebastian G. Michel

**Affiliations:** ^1^ Department of Cardiac Surgery, Ludwig Maximilian University Munich, Munich, Germany; ^2^ Department of Pediatric Cardiology and Intensive Care, Ludwig Maximilian University Munich, Munich, Germany; ^3^ Munich Heart Alliance, German Center for Cardiovascular Research (DZHK), Munich, Germany; ^4^ Comprehensive Pneumology Center Munich, German Center for Lung Research (DZL), Munich, Germany; ^5^ Department of Medicine V, Pulmonology, Ludwig Maximilian University Munich, Munich, Germany; ^6^ Department of Anesthesiology, Ludwig Maximilian University Munich, Munich, Germany; ^7^ Department of Thoracic and Cardiovascular Surgery, Heart and Diabetes Center North Rhine-Westphalia, Bad Oeynhausen, Germany; ^8^ Division of Congenital and Pediatric Heart Surgery, Department of Cardiac Surgery, Ludwig Maximilian University Munich, Munich, Germany; ^9^ Department of Congenital and Pediatric Heart Surgery, German Heart Center Munich, Technical University of Munich, Munich, Germany

**Keywords:** hickmann-catheter, pulmonary embolism, chronic thromboembolic pulmonary hypertension, pulmonary endarterectomy, parenteral nutrition (PN)

## Abstract

Chronic thromboembolic pulmonary hypertension is a rare but life-threatening complication of long-term central venous catheters (CVC) in children. However, evidence in terms of potential treatment strategies and outcome data remains scarce. We describe two cases of CVC-related thrombosis (Hickman-catheter) complicated by recurrent pulmonary emboli. One patient experienced a complete thromboembolic obstruction of the right pulmonary artery with normal pulmonary pressures and the second patient suffered from a central thromboembolic obstruction of both pulmonary arteries associated with severe pulmonary hypertension. Both patients successfully underwent surgical thromboendarterectomy with deep hypothermic circulatory arrest.

## Introduction

Central venous catheter (CVC) thrombosis complicated by pulmonary emboli (PE) is a clinically underrecognized and possibly life-threatening complication of long-term central venous access (1–5). Children requiring parenteral nutrition (PN) are particularly affected due to multifactorial causes and the prevalence of PE in this specific cohort is up to 32% (6–8). Clinical symptoms can be non-specific or even absent and therefore diagnosis and initiation of anticoagulant therapy are often delayed (3). This poses an increased risk for incomplete thrombus resolution, fibrotic remodeling and small vessel-disease, ultimately leading to chronic thromboembolic pulmonary hypertension (CTEPH) (9, 10). If left untreated, patients will inevitably experience right ventricular failure due to increased pulmonary pressures and resistance. Therefore, pulmonary thromboendarterectomy (PTE) is the treatment of choice in adult patients (10). Whereas PTE is associated with excellent short and mid-term results (1-year survival 93% and 3-year survival 89%) in the adult population (11), data for pediatric CTEPH patients is deemed insufficient as they roughly present 1% of all CTEPH cases (4, 5, 12, 13). Herein, we present two pediatric patients who received a Hickman-catheter for parenteral nutrition and developed CVC-related thrombosis complicated by PE. The first patient suffered from chronic PE, in which pulmonary pressures remained within normal range and the second patient experienced severe CTEPH. Both patients were successfully treated with surgical PTE with deep hypothermic circulatory arrest.

## Case 1

**Figure 1:**
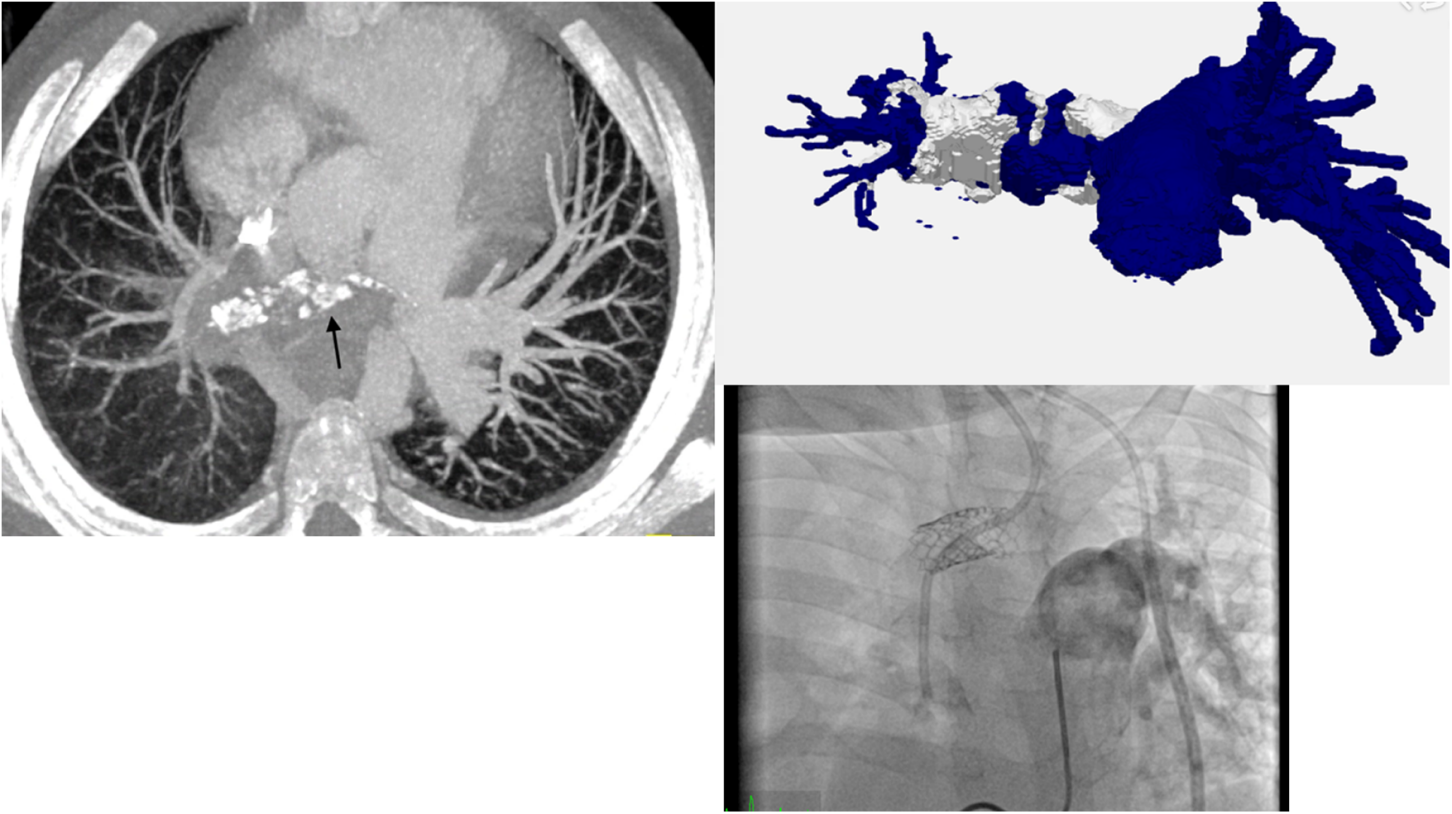
CT-angiography, 3D-reconstruction and pulmonary angiogram showing subtotal obstruction of the right pulmonary artery (patient 1).

**Figure 2:**
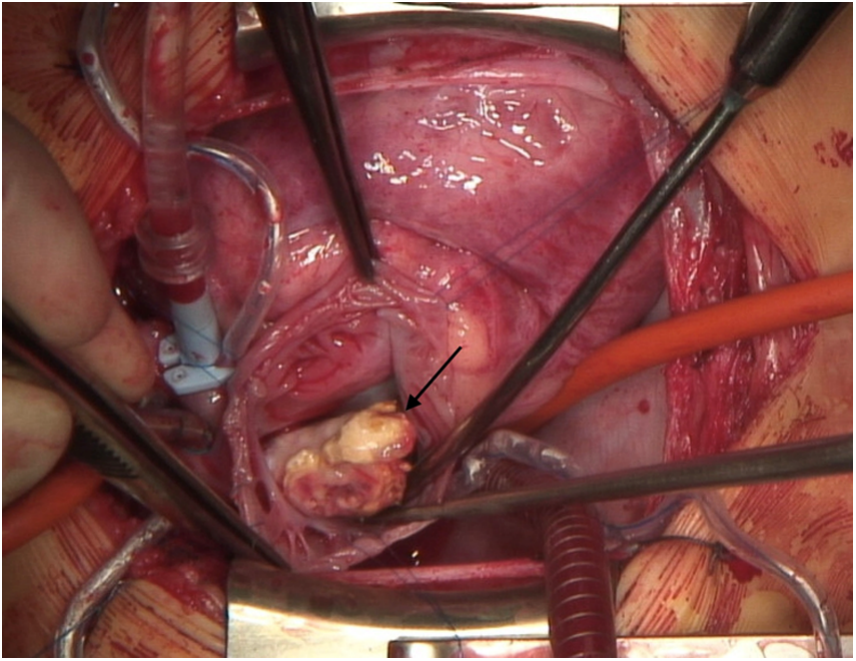
Visible thrombus in the **right** atrium after atriotomy (patient 1).

The first patient was a 6-year-old male with an immune dysregulation disorder. It was most likely attributed to a mutation of the acyloxyacyl hydrolase gene. The disorder manifested as Crohn's like disease with bloody diarrhea at age two, which resulted in several episodes of ileus and subileus. A Hickman-catheter was placed in the left jugular vein at age two for PN. In the following year, the CVC was exchanged 3 times due to recurrent CVC-related sepsis (Candida albicans and Enterococcus faecium). Additionally, he developed a thrombotic occlusion of the brachiocephalic vein, which was resolved by stenting. At age 5, routine echocardiography revealed a thrombus at the tip of the catheter in the right atrium. The patient was asymptomatic and was treated with Phenprocoumon (target INR 2.5–3.5) for 1 year. Follow-up echocardiography showed a persistent thrombus size of 3.3 cm × 1.7 cm. CT-angiography revealed a total thromboembolic obstruction of the right pulmonary artery and a partial obstruction of the left pulmonary artery (Figure 1). At that time, the patient remained clinically stable, right ventricular function was intact and pulmonary pressures were within normal range (mean pulmonary artery pressure: 21 mmHg). Nevertheless, the decision for PTE was made because of evident disease progression despite the administration of oral anticoagulation. After sternotomy and bicaval cannulation, cardiopulmonary bypass with deep hypothermic circulatory arrest (18°C) was established. First, right atriotomy was performed (Figure 2). The thrombus was removed, and the Hickman-catheter was shortened at the level of the vena cava superior. Subsequently, the right pulmonary artery was incised and the thrombotic material was completely extracted and the vessel endarterectomized. Histologic analysis revealed necrotic, partially calcified thrombotic material without signs of a malignant or infective process. The CVC was left *in situ* because of the difficult venous access and status post stenting of the brachiocephalic vein. The patient was extubated on the first postoperative day and the early clinical course was satisfactory. Three weeks later, he underwent subxiphoid pericardiostomy due to the accumulation of pericardial effusion. Two months after surgery, a single rethoracotomy was performed because of deep sternal wound infection and mediastinitis. Micriobiological analysis identified staphylococcus aureus as the causative agent. The remaining clinical course was uneventful and the anticoagulation regime included subcutaneous administration of Enoxaparin natrium (target anti-Xa 0.8–1.2 IU/ml) for 12 months. The Hickman-catheter was removed 3 years later. No further thromboembolic events occurred within 5 years after surgery.

## Case 2

**Figure 3:**
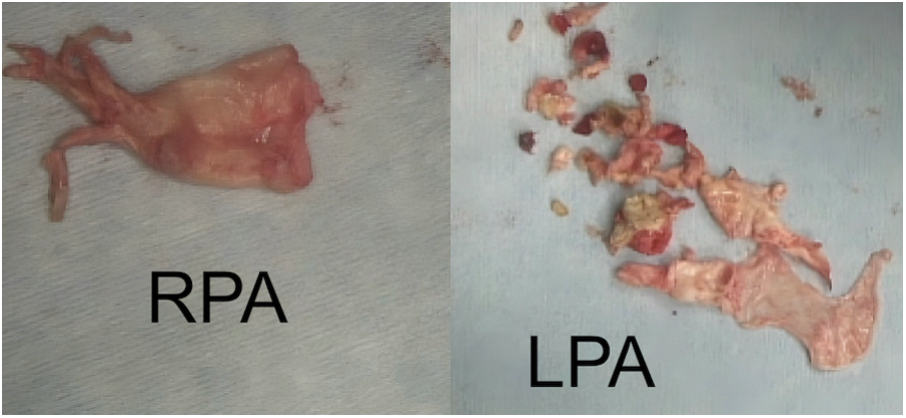
Removed thrombotic material from both pulmonary arteries (patient 2). RPA, **right** pulmonary artery; LPA, **left** pulmonary artery.

CVC, central venous catheter; PTE, pulmonary thromboendarterectomy; RPA, right pulmonary artery; LPA, left pulmonary artery.

The second patient was a 7-year-old male, who suffered from short bowel syndrome as a consequence of multiple abdominal injuries and received a Hickman-catheter for PN through the left internal jugular vein. He experienced reduced exertional capacity and dyspnea, which was initially interpreted as an asthma exacerbation and was treated as such. Nonetheless, symptoms progressed to dyspnea at rest and the patient presented with mild hypoxemia. Therefore, a cardiac workup was performed as well. Echocardiography revealed a slightly reduced right ventricular function (tricuspid annular plane systolic excursion: 16 mm), right ventricular dilation (right ventricular end diastolic diameter 27 mm) with hypertrophic changes, a moderate insufficiency of the tricuspid valve and signs of pulmonary hypertension (systolic pulmonary pressure 50 mmHg + central venous pressure). Furthermore, a calcified thrombus at the tip of the Hickman-catheter in the right atrium (1.0 cm × 1.4 cm) and a reduced flow in both pulmonary arteries were detected. CT-angiography showed a central PE with progression into both pulmonary arteries. Therefore, anticoagulation with subcutaneous Enoxaparin natrium (target anti-Xa 0.8–1.0). was initiated. At first, clinical recompensation could be achieved. In the following three months, the right atrial thrombus grew in size (3.2 cm × 1.6 cm) despite anticoagulant therapy and consequently, the patient underwent two cycles of systemic thrombolytic therapy with Alteplase. As it did not lead to thrombus resolution and the patient's pulmonary function progressively declined requiring oxygen therapy (up to 6 L/min), interventional balloon angioplasty with intravascular lithiolysis was performed (14). However, it only resulted in a minimally improved pulmonary artery flow and pulmonary pressures remained significantly increased (mean pulmonary artery pressure: 57 mmHg). The decision for PTE was made. The procedure was the same as described in the first case but this time, the Hickman-catheter was completely removed and both pulmonary arteries were completely endarterectomized (Figure 3). The patient was extubated on the second postoperative day, pulmonary pressures quickly decreased to normal values and he was referred to another hospital on the sixth day after surgery. The subsequent postoperative course was uneventful, but the patient required prolonged neurologic rehabilitation. The child was treated with Enoxaparin natrium for 6 months.

## Discussion

Patients with CTEPH present a small subgroup (<1%) of the general pediatric population suffering from pulmonary hypertension and as specific treatment recommendations are non-existing, therapeutic approaches are still based on guidelines for adult CTEPH patients (10, 15). We describe two rare cases of CVC-related CTEPH in children suffering from gastrointestinal failure.

Patients requiring long-term PN are prone to thromboembolic events on account of low levels of natural anticoagulants (antithrombin, protein S and protein C) as well as procoagulatory properties of the PN solution, namely crystal precipitation of amino acids and calcium, platelet activation, dextrose favoring hypercoagulation and calcium-mediated activation of the coagulation cascade (6–8). The catheter tip position presents an additional factor. In the described cases, the catheter tip was located in the right atrium, which has been associated with CVC-related atrial thrombosis in the past due to simple mechanical irritation of the atrial wall and non-physiological blood flow patterns (16, 17). The first patient also had a history of CVC-related sepsis, a known risk factor for CVC-related thrombosis as well (1, 8). Because of the increased thrombotic risk, discussions regarding prophylactic anticoagulation in this specific patient cohort are still ongoing. Our patients were treated in accordance with current guidelines, in which secondary prophylaxis is preferred and anticoagulation as a primary preventive measure is not recommended (18, 19). However, two smaller studies, investigating children receiving long-term PN, suggest that prophylactic anticoagulation effectively reduces CVC-related thrombosis with low rates of bleeding complications and thus may be considered in high-risk patients (20, 21).


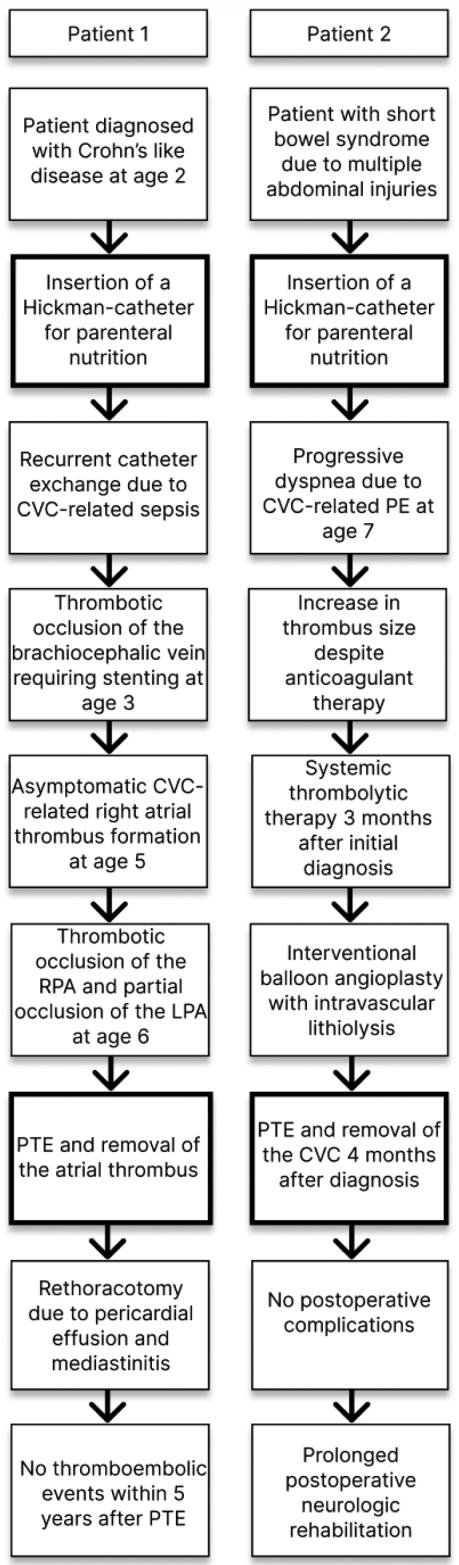


The optimal treatment for PE due to CVC-related thrombosis is still unknown, but a conservative approach in terms of anticoagulant and systemic thrombolytic therapy has been recently proposed for children on chronic haemodialysis (22). This strategy neither lead to atrial thrombus resolution, nor restoration of pulmonary circulation in both of the presented patients. Most likely, because our patients were (typically) diagnosed at an advanced stage with extensive and partially calcified thrombotic material unlikely to dissolve, and consequently underwent surgical PTE.

Comparable publications are limited to a small number of case reports. Lambert et al. described the first case of CTEPH in an infant related to a Broviac-catheter in 1999, who underwent PTE after a failed attempt of systemic thrombolytic therapy (23). Spencer et al. presented a case of CTEPH in an 11-year-old boy with sickle cell disease and a history of CVC-related thrombosis, who was primarily unsuccessfully treated with anticoagulant therapy and PTE was therefore performed in the later clinical course (4). Humpl et al. reported on a case of PTE after ineffective anticoagulation in a 16-month-old girl suffering from CTEPH associated with a peritoneovenous Denver shunt (5). Verbelen et al. presented the clinical course of a 12-year-old boy on permanent parenteral nutrition and frequent infection-related port-a-cath exchanges developing CTEPH (24). All of these patients, including ours, were critically ill and required long-term CVC-placement. They were successfully treated with PTE followed by an 100% survival to hospital discharge. The case series (17 patients) by Madani et al. supports these findings, in which PTE for CTEPH resulted in a 88% survival rate at 5-years post-surgery (12).

This article highlights the necessity of increased awareness for CVC-related thrombosis as a potential cause of CTEPH and the extended life spans of critically ill pediatric patients with greater thrombotic risk, including the requirement of long-term CVC-placement, could lead to a further increase in pediatric CTEPH patients. In conclusion, surgical PTE is a safe and feasible procedure in children suffering from CVC-related CTEPH and should not be delayed in favor of anticoagulation or systemic thrombolytic therapy. With an increasing number of such reports recently, routine echocardiographic screening for CVC-related thrombosis and potential signs of pulmonary hypertension should be performed to facilitate early detection of intracardiac thrombotic material as a potential cause for CTEPH.

## Data Availability

The original contributions presented in the study are included in the article, further inquiries can be directed to the corresponding author.
